# Effect of histological breast cancer subtypes invasive lobular versus non-special type on survival in early intermediate-to-high-risk breast carcinoma: results from the SUCCESS trials

**DOI:** 10.1186/s13058-023-01750-0

**Published:** 2023-12-14

**Authors:** Davut Dayan, Stefan Lukac, Brigitte Rack, Florian Ebner, Visnja Fink, Elena Leinert, Kristina Veselinovic, Sabine Schütze, Ziad El Taie, Wolfgang Janni, Thomas W. P. Friedl

**Affiliations:** 1 Department of Gynecology and Obstetrics, University of Ulm, Prittwitzstraße 43, 89075 Ulm, Germany; 2 Gyn-Freising, Freising, Germany

**Keywords:** Breast cancer, Invasive lobular carcinoma, No special type carcinoma, Survival, Lymph node infiltration

## Abstract

**Background:**

Invasive lobular breast carcinomas (ILC) have different histological features compared to non-special type carcinomas (NST), but the effect of histological subtypes on survival is controversial. In this study, we compared clinicopathological characteristics and outcomes between ILC and NST based on a large pooled data set from three adjuvant breast cancer trials (SUCCESS A, B, and C) and investigated a potential differential effect of recurrence risk related to nodal stage on survival.

**Methods:**

From 2005 to 2017, the large randomized controlled SUCCESS A, B, and C trials enrolled 8190 patients with primary, intermediate-to-high-risk breast carcinoma. All patients received adjuvant chemotherapy, and endocrine and/or HER2-targeted treatment was given where appropriate. Survival outcomes in terms of disease-free survival (DFS), overall survival (OS), breast cancer-specific survival (BCSS), and distant disease-free survival (DDFS) were estimated using the Kaplan–Meier method and analyzed using log-rank tests as well as univariable and adjusted multivariable Cox regression models.

**Results:**

In the SUCCESS trials, 6284 patients had NST and 952 had ILC. The median follow-up time was 64 months. ILC patients were older, more likely to receive mastectomy, and more likely to have larger tumor sizes, lymph node infiltration, hormone receptor-positive, HER2neu-negative, and luminal A-like tumors than NST patients. In the overall cohort, no significant differences between ILC and NST were detectable regarding the four survival endpoints, with hazard ratios obtained in adjusted multivariable cox regressions of 0.96 (95% CI 0.77–1.21, *p* = 0.743) for DFS, 1.13 (95% CI 0.85–1.50, *p* = 0.414) for OS, 1.21 (95% CI 0.89–1.66, *p* = 0.229) for BCSS, and 0.95 (95% CI 0.73–1.24, *p* = 0.689) for DDFS. However, a differential effect of nodal stage on survival was observed, with better survival for ILC patients with pN0/pN1 tumors and worse survival for ILC patients with pN2/pN3 tumors compared to NST patients.

**Conclusions:**

Our results revealed that ILC was associated with worse survival compared to NST for patients at high risk of recurrence due to advanced lymph node infiltration. These findings should be taken into account for treatment decisions and monitoring.

## Introduction

Invasive lobular carcinoma (ILC) is the second most common breast cancer subtype after non-special type carcinoma (NST), which was formerly known as invasive ductal carcinoma (IDC). ILC accounts for 10–15% of all breast cancers [1–3]. It displays different clinical and biological features to NST, notably the absence of the cell adhesion molecule E-cadherin [3–6]. Despite slow growth, ILC is more likely than NST to be detected at higher stages due to a presentation of architectural distortion and poor imaging sensitivity [2, 3, 7–10]. More often than NST, ILC tends to be multifocal, hormone receptor (HR)-positive, HER2neu-negative, and low grade (GI or II) [4, 11]. It also shows a reduced response rate to neoadjuvant chemotherapy (CHT) [4, 12–15] and may respond differentially to adjuvant endocrine therapies than NST. The therapeutic benefit obtained through treatment with aromatase inhibitors (AIs) compared to treatment with tamoxifen seems to be greater in ILC than in NST [16–18].

Grading, tumor size, lymph node infiltration, and positive resection margins are well-known prognostic factors of breast cancer. With regard to the prognostic effect of histological tumor subtype, studies show conflicting results. While some studies show higher risk of recurrence and poor prognosis in ILC [8, 12, 13, 19–21], other studies show similar or even better prognosis of ILC compared to NST [5, 22, 23].

We performed a pooled analysis of three large randomized phase III adjuvant breast cancer trials (SUCCESS A, B, & C) to analyze the effect of histological tumor subtype on prognosis. In addition, we investigated whether there was a differential prognostic effect of histological tumor subtype on survival depending on nodal involvement, i.e., whether a comparison of survival outcome between patients with ILC and NST yielded different results depending on nodal status. Several retrospective studies have provided outcome data for various subgroups, and a recent study found different prognostic outcomes of ILC and NST depending on molecular subtypes [22]. However, to our knowledge, an interaction effect between histological type and nodal status has not been investigated in detail before. Such findings could have an immediate impact on the adjuvant treatment recommendations.

## Material and methods

### SUCCESS studies

The SUCCESS studies are a series of three consecutive clinical trials conducted between 2005 and 2017 on primary intermediate-to-high-risk breast cancer patients. The study series comprises the SUCCESS A trial ("**S**imultaneous St**u**dy of Gem**c**itabine-Docetaxel **C**ombination adjuvant treatment, as well as **E**xtended Bi**s**phosphonate and **S**urveillance-Trial"; NCT02181101), the SUCCESS B trial ("**S**imultaneous St**u**dy of Gem**c**itabine-Docetaxel **C**ombination adjuvant tr**e**atment, a**s** well a**s** Biological Targeted Treatment"; NCT00670878), and the SUCCESS C trial ("**S**imultaneous St**u**dy of Do**c**etaxel-based Anthra**c**ycline-free adjuvant treatment **e**valuation, as well as Life **S**tyle Intervention **S**trategies"; NCT00847444). Overall, 8190 patients were included in the SUCCESS A, B, and C trials. Inclusion criteria were primary R0-resected epithelial invasive breast cancers (pT1-4, pN0-3, pM0) with guideline adherent treatment recommendation for adjuvant CHT (nodal positive or high-risk nodal negative (pT ≥ 2, tumor grade (G) 3, ≤ 35 years old, or negative hormone receptor status—further details in “Appendix”). Patients received obligatory radiotherapy following breast-conserving surgery or ≥ 4 axillary lymph node metastases; for patients undergoing mastectomy radiotherapy was also recommended in case of tumors > 3 cm and, in case of 1–3 involved lymph nodes and the presence of at least one of the additional risk factors multicentric growth, lymphangiosis or hemangiosis carcinomatosa, pectoralis fascia involvement, or a safety margin of < 5 mm. Adjuvant endocrine therapy was administered according to guideline standards for premenopausal and postmenopausal patients with hormone receptor positive tumors. The first follow-up was performed 4 weeks after the last course of chemotherapy and 6 weeks after the last administration of radiotherapy. Further controls were analogous to the usual follow-up of breast carcinoma: every 3 months for the first 3 years and every 6 months during the following 2 years [24, 25].

The Federal Institute for Drugs and Medical Devices (BfArM) and the relevant ethics committees approved the SUCCESS studies (Success A: Ludwig Maximilian University Munich, 076/05; Success B: Ludwig Maximilian University Munich, 395/07; Success C: Heinrich-Heine University Duesseldorf, MC-LKP-319), which were performed in accordance with Good Clinical Practice and the Declaration of Helsinki. Written informed consent was obtained from all patients.

### SUCCESS A

This open-label, multicenter, 1:1 randomized phase III study was conducted with 3754 primary intermediate-to-high-risk breast cancer patients at 271 study sites. Participants were recruited between September 2005 and March 2007. The primary objective was to compare disease-free survival after adjuvant CHT with or without gemcitabine. All patients received three cycles of FEC (fluorouracil, epirubicin, cyclophosphamide; 100/500/500 mg/m^2^) followed by either three cycles of docetaxel alone (100 mg/m^2^, q3w) (FEC-Doc) or gemcitabine (1000 mg/m^2^ on days 1 and 8) and docetaxel (75 mg/m^2^, q3w) (FEC-DocG). The second randomization was after completion of CHT to assess disease-free survival with 2 years of zoledronate treatment (every 3 months for 24 months) versus 5 years of zoledronate treatment (every 3 months for 24 months, followed by every 6 months for 36 months). If adjuvant endocrine therapy was indicated, premenopausal patients were administered with tamoxifen 20 mg q1d p.o. (and if they were < 40 years, they were additionally administered with goserelin 3.6 mg q4w s.c.); postmenopausal patients were administered with tamoxifen for 2 years followed by 3 years of anastrozole 1 mg q1d p.o. Important secondary objectives were assessment of overall survival, distant disease-free survival, toxicity, quality of life, and skeletal morbidity.

### SUCCESS B

SUCCESS B was another open-label, multicenter, 1:1 randomized phase III study that enrolled 793 intermediate-to-high-risk breast cancer patients between June 2008 and September 2011. Study participants were positive for human epidermal growth factor receptors (HER2neu) and received the same adjuvant CHT as in the SUCCESS A study (i.e., FEC-Doc vs FEC-DocG). Following CHT, all patients received HER2neu targeted therapy with trastuzumab (8 mg/kg intravenous (IV) loading dose followed by 6 mg/kg IV every 3 weeks for a total of 52 weeks). The primary study objective was to compare disease-free survival between the two randomization arms. The secondary objectives were to compare overall survival, distant disease-free survival, toxicity, and change in quality of life. If adjuvant endocrine therapy was indicated, patients received 5 years of tamoxifen 20 mg q1d p.o. plus goserelin 3.6 mg q4w s.c. (for premenopausal patients) or 5 years of letrozole 2.5 mg q1d p.o. (for postmenopausal patients). In contrast to SUCCESS A, the use of adjuvant bisphosphonates was allowed for primary prophylactic treatment but was not generally recommended.

### SUCCESS C

The SUCCESS C trial was a multicenter, prospective randomized phase III study comprising 3643 patients with primary HER2neu negative early breast cancer that were enrolled between February 2009 and August 2011 at 231 German study sites. The first randomization examined disease-free survival in patients receiving either anthracycline-free CHT treatment (6 cycles of docetaxel-cyclophosphamide, Doc-C) or anthracycline-containing CHT treatment with FEC-Doc. The second randomization compared disease-free survival in patients with a body mass index (BMI) of 24–40 kg/m^2^ who received either an individualized lifestyle intervention program or general healthy lifestyle recommendations. The individualized lifestyle intervention program comprised a two-year standardized and structured telephone intervention aimed at moderate weight loss through dietary changes and physical activity, supported by educational materials and patient diary. If adjuvant endocrine therapy was indicated, patients received 5-year treatment with tamoxifen 20 mg q1d p.o. and goserelin 3.6 mg q4w s.c. if indicated or exemestane 25 mg q1d p.o. As in the Success B study but in contrast to the SUCCESS A study, the use of adjuvant bisphosphonates was allowed but not generally recommended.

### Clinicopathological parameters

We categorized age as ≤ 50 years, 51–65 years and > 65 years and BMI according to the World Health Organization (WHO) classification into the categories < 18.5 kg/m^2^ (underweight), 18.5–24.9 kg/m^2^ (normal weight), 25.0–29.9 kg/m^2^ (overweight) and ≥ 30.0 kg/m^2^ (obese). Primary tumor stage (pT1-4) and nodal status (pN0-3) were defined using the American Joint Committee on Cancer and International Association Against Cancer (UICC) revised TNM classification system criteria [26].

Histological grading (G1, G2, G3) was performed according to Elston–Ellis modification with Scarff–Bloom–Richardson criteria [27]. For hormone receptor status, tumors were graded as positive or negative. Tumors were classified as hormone receptor positive if ≥ 10% of the cells in the tumor tissue had estrogen and/or progesterone receptors. Tumors were classified as HER2neu positive if they had strong immunohistochemical amplifications of HER2neu receptors (3+) or if they had moderate immunohistochemical amplifications of HER2neu receptors (2+) and a positive result in FISH analysis. Biological tumor subtypes were defined in the absence of consistent Ki-67 determinations as follows. Luminal A-like: hormone receptor positive, HER2neu negative, G1-2; Luminal B-like: hormone receptor positive, HER2neu negative, G3; HER2 type: HER2neu positive; triple negative breast carcinoma: hormone receptor negative, HER2neu negative [28–31].

HER2neu targeted therapy comprised mainly, but not exclusively, treatment with trastuzumab, asrastuzumab was approved in the adjuvant setting in 2006 during the recruitment of the SUCCESS A study. Endocrine therapy was not documented consistently for all patients, as some patients received their endocrine therapy at their general practitioners or gynecologists practice rather than at the study center, not always providing this information to the study center for documentation purposes. Thus, some of these patients may have been misclassified as having received no endocrine therapy.

Tumor classification into histological type (ductal, lobular, other) was based on the current WHO histopathologic classification [32, 33].

### Data analysis

All categorical data are described in terms of absolute and relative frequencies, whereas the continuous variables, age and BMI, are additionally described by reporting mean ± SD, median, and range. Associations of patient or tumor characteristics with histological type (NST and ILC) were assessed using Chi-square tests for all categorical variables and with Mann–Whitney U tests for the continuous variables age and BMI. As we were also interested in possible interaction effects involving nodal status (see below), we categorized nodal status into the two categories pN0/pN1 and pN2/pN3 for all subsequent analyses to facilitate 2-way interaction tests while retaining meaningful sample sizes for the analyzed subgroups.

Survival parameters were analyzed using the Kaplan–Meier method and summarized with medians and 95% confidence intervals, and survival curves were compared using log-rank tests. Survival times were measured from the date of randomization to the date of the event or, if no endpoint was reached, to the data of last appropriate follow-up (censoring). Four different survival endpoints defined according to the Standardized Definitions of Efficacy Endpoints (STEEP) criteria were analyzed [34]. Disease-free survival (DFS) included local, contralateral, and distant metastatic disease, secondary primary tumors, and death from any cause as an event. Distant disease-free survival (DDFS) included only distant recurrences (metastases and secondary primary tumors) and death from any cause as events; ipsilateral or regional disease recurrences and contralateral breast cancer were excluded. Overall survival (OS) included death from any cause as an event. Breast cancer-specific survival (BCSS) included only deaths from breast cancer-related causes (e.g., metastasis-related organ failure or breast cancer progression) as an event, while patients that died for other reasons were censored at the date of death.

To evaluate whether the histological tumor type (NST, ILC) is an independent prognostic factor, we used multivariable Cox proportional hazards regression models adjusted for age (≤ 50, 51–65, > 65), BMI (underweight, normal weight, overweight, obese), tumor size (pT1, pT2, pT3, pT4), nodal stage (pN0/pN1, pN2/pN3), tumor grade (G1, G2, G3), hormone receptor status (positive, negative), HER2 status (positive, negative), menopausal status (premenopausal, postmenopausal), type of surgery (breast conserving, mastectomy, other), chemotherapy treatment (FEC-Doc, FEC-DocG, Doc-C), endocrine therapy (yes, no), radiotherapy (yes, no), and bisphosphonate therapy (yes, no). Please note that we have not adjusted for duration of bisphosphonate treatment, as a comprehensive analysis of the randomized SUCCESS A trial revealed no difference in survival between 2 and 5 years of adjuvant bisphosphonate (zoledronate) treatment [35].

To investigate whether the effect of histological tumor type on survival was influenced by nodal stage, we first ran Cox regression models with the main effects of histological tumor type and nodal stage together with the two-way interaction term between histological tumor type and nodal stage for all four survival endpoints. If the two-way interaction was significant, we subsequently ran a multivariable fully adjusted Cox regression model to test whether the two-way interaction term remained significant when all main effects of known prognostic factors were accounted for.

Statistical analyses were performed using IBM® SPSS® statistics, version 24 (IBM Corp., Armonk, NY, USA). All statistical tests were two-sided, and *p* values < 0.05 were considered significant. There was no adjustment for significance level for multiple testing.

## Results

In our analysis, we were able to include 7236 cases out of 8190 SUCCESS patients (6284 cases with an invasive ductal breast cancer (NST) and 952 cases with an invasive lobular breast cancer). The remaining 954 patients had either an unknown tumor histology (*n* = 24) or other invasive epithelial breast carcinomas (*n* = 930) and were excluded from the analysis.

### Comparison of clinicopathological parameters between ILC and NST

The median age of the 7236 study participants was 54 years (range 22–86 years). Table 1 shows the comparison of clinicopathological factors between patients with NST (*n* = 6284) or ILC (*n* = 952) tumors. Significant differences between NST and ILC were found with regard to all parameters investigated, except BMI. NST patients were more likely to be < 50 years old and more often premenopausal compared to ILC patients (39.1% vs. 28.7% and 41.3% vs. 32.9%, respectively). Breast-conserving surgical treatment was performed more often on the NST tumors (75.5% vs. 54.0%); accordingly, ILC patients were significantly less likely to receive adjuvant radiotherapy (80.7% vs. 85.3%).

**Table 1:** Baseline patient, tumor, and treatment characteristics according to histological tumor type

	Total (*n* = 7236)		NST (*n* = 6284)		ILC (*n* = 952)		*P* value*
	*n*	%	*n*	%	*n*	%	
*Study*							< 0.001
SUCCESS A	3479	48,1	3060	48.7	419	44.0	
SUCCESS B (HER2neu positive only)	320	4.4	294	4.7	26	2.7	
SUCCESS C (HER2neu negative only)	3437	47.5	2930	46.6	507	53.3	
*Age (years)*							< 0.001
Mean ± SD	54.3 ± 10.3		53.9 ± 10.4		56.7 ± 9.3		
Median value	54.0		54.0		57.0		
Range	22–86		22–86		29–85		
*Age category (years)*							< 0.001
≤ 50	2733	37.8	2460	39.1	273	28.7	
51–65	3274	45.2	2785	44.3	489	51.4	
> 65	1229	17.0	1039	16.5	190	20.0	
*BMI (kg/m*^*2*^*)*							0.164
Mean ± SD	26.5		26.5 ± 5.2		26.6 ± 4.9		
Median value	25.5		25.5		2.6		
Range	15.4–53.9		15.4–53.9		16.6–48.7		
*BMI category (kg/m*^*2*^*)*							0.670
< 18.5	87	1.2	74	1.2	13	1.4	
18.5–24.99	3227	44.6	2818	44.8	409	43.0	
25–29.99	2315	32.0	2007	31.9	308	32.4	
≥ 30	1607	22.2	1385	22.0	222	23.3	
*Menopausal status*							< 0.001
Premenopausal	2911	40.2	2598	41.3	313	32.9	
Postmenopausal	4325	59.8	3686	58.7	639	67.1	
*Surgical treatment*							< 0.001
BET	5257	72.7	4743	75.5	514	54.0	
Mastectomy	1800	24.9	1389	22.1	411	43.2	
Other	179	2.5	152	2.4	27	2.8	
*Tumor size (pT)*							< 0.001
pT1	3117	43.1	2858	45.5	259	27.2	
pT2	3659	50.6	3139	50.0	520	54.6	
pT3	361	5.0	205	3.3	156	16.4	
pT4	96	1.3	79	1.3	17	1.8	
Missing	3	0.0	3	0.0	0	0.0	
*Nodal status (pN)*							< 0.001
pN0	2746	37.9	2454	39.1	292	30.7	
pN1	3343	46.2	2904	46.2	439	46.1	
pN2	805	11.1	682	10.9	123	12.9	
pN3	332	4.6	235	3.7	97	10.2	
Missing	10	0.1	9	0.1	1	0.1	
*Nodal status category (pN)*							< 0.001
pN0/pN1	6089	84.1	5358	85.3	731	76.8	
pN2/pN3	1137	15.7	917	14.6	220	23.1	
Missing	10	0.1	9	0.1	1	0.1	
*Hormone receptor (HR) status*							< 0.001
Negative (ER and PR negative)	1873	25.9	1815	28.9	58	6.1	
Positive (ER and/or PR positive)	5362	74.1	4468	71.1	894	93.9	
Missing	1	0.0	1	0.0	0	0.0	
*Estrogen receptor (ER) status*							< 0.001
Negative	2070	28.6	1990	31.7	80	8.4	
Positive	5163	71.4	4292	68.3	871	91.5	
Missing	3	0.0	2	0.0	1	0.1	
*Progesterone receptor (PR) status*							< 0.001
Negative	2599	35.9	2435	38.7	164	17.2	
Positive	4633	64.0	3846	61.2	787	82.7	
Missing	4	0.1	3	0.0	1	0.1	
*HER2neu-status*							< 0.001
Negative	6007	83.0	5133	81.7	874	91.8	
Positive	1166	16.1	1095	17.4	71	7.5	
Missing	63	0.9	56	0.9	7	0.7	
*Grading*							< 0.001
G1	379	5.2	346	5.5	33	3.5	
G2	3595	49.7	2852	45.4	743	78.0	
G3	3259	45.0	3085	49.1	174	18.3	
Missing	3	0.0	1	0.0	2	0.2	
*Biological subtype*							< 0.001
Luminal A (HR+/HER2neu−/G1-2)	3194	44.1	2489	39.6	705	74.1	
Luminal B (HR+/HER2neu−/G3)	1386	19.2	1258	20.0	128	13.4	
HER2neu type (HER2neu+)	1166	16.1	1095	17.4	71	7.5	
Triple negative (HR-/HER2neu-)	1426	19.7	1386	22.1	40	4.2	
Missing	64	0.9	56	0.9	8	0.8	
*Chemotherapy*							0.005
FEC-Doc (all SUCCESS studies)	3628	50.1	3153	50.2	475	49.9	
FEC-DocG (SUCCESS A and B)	1884	26.0	1668	26.5	216	22.7	
Doc-C (SUCCESS C only)	1724	23.8	1463	23.3	261	27.4	
*Endocrine therapy*							0.008
No	3956	54.7	3476	55.3	480	50.4	
Yes	3267	45.1	2801	44.6	466	48.9	
Missing	13	0.2	7	0.1	6	0.6	
*HER2-targeted therapy*							< 0.001
No	6384	88.2	5483	87.3	901	94.6	
Yes	839	11.6	794	12.6	45	4.7	
Missing	13	0.2	7	0.1	6	0.6	
*Radiotherapy*							< 0.001
No	1096	15.1	918	14.6	178	18.7	
Yes	6127	84.7	5359	85.3	768	80.7	
Missing	13	0.2	7	0.1	6	0.6	
*Bisphosphonate therapy*							0.016
No	3876	53.6	3334	53.1	542	56.9	
Yes	3347	46.3	2943	46.8	404	42.4	
Missing	13	0.2	7	0.1	6	0.6	

*FEC* fluorouracil, epirubicin, cyclophosphamide, *DOC-G* docetaxel with gemcitabine, *DOC-C* docetaxel with cyclophosphamide

*Chi-square tests for categorical variables; Mann–Whitney U tests for continuous metric variables; all tests without missings

ILC tumors were diagnosed in more advanced tumor stages (18.2% vs. 4.5% pT3/pT4 tumors) and with more frequent lymph node infiltration (23.1% vs. 14.6% pN2/pN3 tumors) compared to NST. However, ILC tumors showed poorly differentiated tumors with grading G3 less often than NST tumors (18.3% vs. 49.1%). The ILC histological type was associated with a higher rate of hormone receptor positive tumors (93.9% vs. 71.1%) than NST. Accordingly, ILC tumors were more often of the luminal subtype (87.5% vs. 59.6%), while the biological subtypes HER2neu positive and triple negative were observed less often compared to NST tumors (7.5% vs. 17.4% and 4.2% vs. 22.1%). For more data and results concerning the comparison of clinicopathological factors between ILC and NST tumors, see Table 1.

#### Survival according to histological type: comparison between ILC and NST tumors

**Fig. 1:**
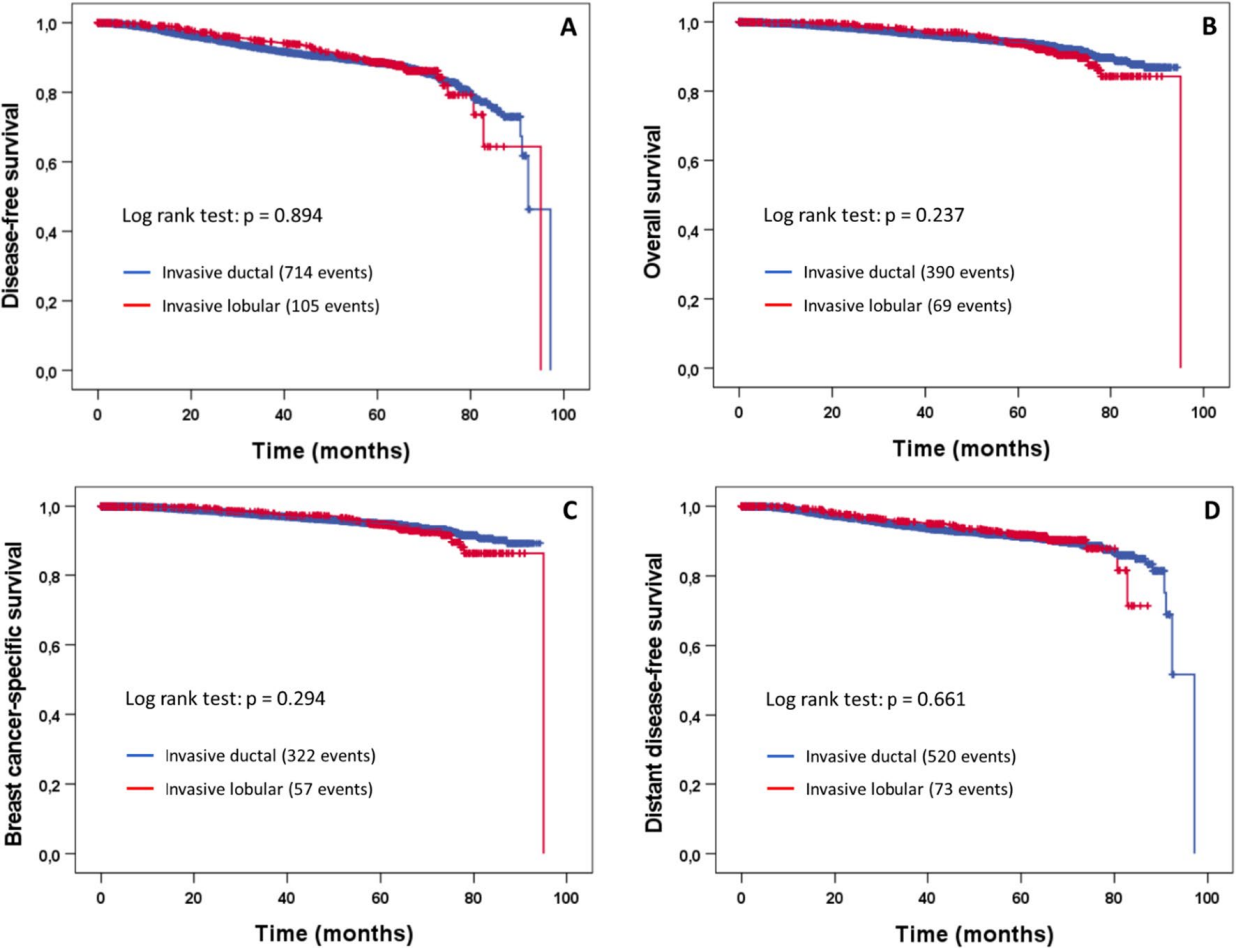
Kaplan–Meier plots of disease-free survival (**A**), overall survival (**B**), breast cancer-specific survival (**C**), and distant disease-free survival (**D**) according to histological tumor type

The median follow-up time was 64.4 months (range 0–95 months). There were no significant differences between NST and ILC tumors with regard to all four survival endpoints analyzed (Fig. 1). The corresponding hazard ratios (ILC vs. NST tumors) obtained in univariable Cox regressions were 0.99 (95% CI 0.80–1.21) for DFS, 1.17 (95% CI 0.90–1.51) for OS, 1.16 (95% CI 0.88–1.55) for BCSS and 0.95 (95% CI 0.74–1.21) for DDFS. Very similar results were found using adjusted multivariable Cox regression models that accounted for the effects of other prognostic factors (DFS: HR 0.96, 95% CI 0.77–1.21, *p* = 0.743; OS: HR 1.13, 95% CI 0.85–1.50, *p* = 0.414; BCSS: HR 1.21, 95% CI 0.89–1.66, *p* = 0.229; DDFS: HR 0.95, 95% CI 0.73–1.24, *p* = 0.689).

#### Differential prognostic effect of histological tumor subtype on survival depending on nodal involvement

**Fig. 2:**
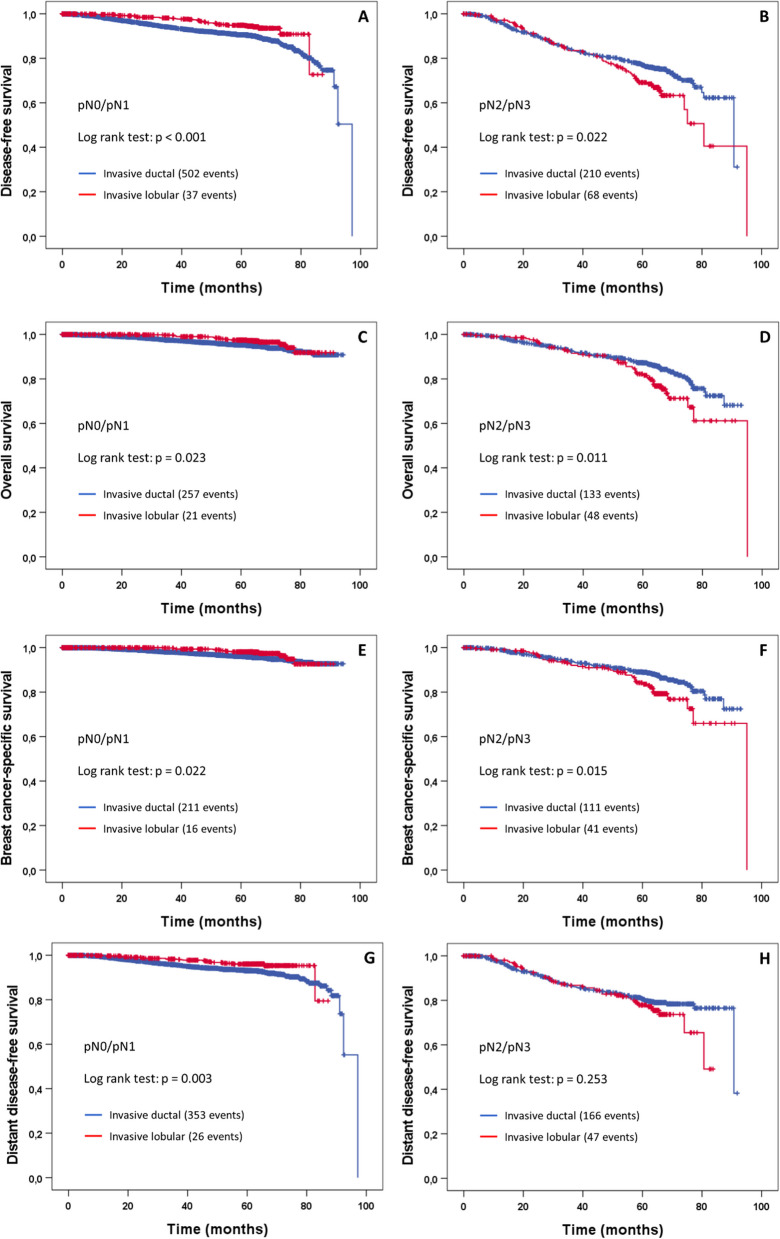
Kaplan–Meier plots of disease-free survival (**A**, **B**), overall survival (**C**, **D**), breast cancer-specific survival (**E**, **F**), and distant disease-free survival (**G**, **H**) according to histological tumor type for pN0/pN1 tumors (left panels) and pN2/pN3 tumors (right panels)

Figure 2 shows the Kaplan–Meier plots for survival of ILC and NST separately for pN0/pN1 and pN2/pN3 tumors. The pattern is very similar among all four survival endpoints. While patients with ILC tumors demonstrate significantly better outcomes compared to patients with NST tumors if lymph node involvement is limited (pN0/pN1), the opposite is true for patients with extended lymph node involvement (pN2/pN3). The corresponding hazard ratios and 95% confidence intervals both for univariable and adjusted multivariable Cox regression models are shown in Table 2.

**Table 2:** Effects of histological tumor type (ILC vs. NST) on DFS, OS, BCSS, and DDFS

Survival endpoint		Univariable Cox regression model			Adjusted multivariable Cox regression model			*p* value for 2-way interaction (histological tumor type × nodal status)
		Hazard ratio	95% CI	*p* value	Hazard ratio	95% CI	*p* value	
DFS	pN0/pN1							< 0.001
	*ILC vs. NST*	0.55	0.39–0.77	< 0.001	0.60	0.42–0.86	0.005	
	pN2/pN3							
	*ILC vs. NST*	1.38	1.05–1.82	0.023	1.40	1.03–1.91	0.033	
OS	pN0/pN1							0.005
	*ILC vs. NST*	0.60	0.39– 0.94	0.025	0.66	0.41–1.07	0.091	
	pN2/pN3							
	*ILC vs. NST*	1.54	1.10–2.15	0.011	1.60	1.09–2.34	0.015	
BCSS	pN0/pN1							0.008
	*ILC vs. NST*	0.56	0.34–0.93	0.024	0.70	0.41–1.20	0.190	
	pN2/pN3							
	*ILC vs. NST*	1.56	1.09–2.24	0.016	1.71	1.12–2.59	0.012	
DDFS	pN0/pN1							0.007
	*ILC vs. NST*	0.55	0.37–0.82	0.003	0.63	0.41–0.95	0.029	
	pN2/pN3							
	*ILC vs. NST*	1.21	0.87–1.67	0.254	1.35	0.94–1.94	0.108	

To analyze in more detail whether the influence of histological tumor type on survival is differentially affected by nodal stage, we calculated the 2-way interactions between histological type (ILC, NST) and nodal stage (pN0/pN1, pN2/pN3) for all four survival endpoints using fully adjusted multivariable Cox regression models. The interaction term was significant for DFS, OS, BCSS, and DDFS (Table 2). These results confirm that the effect of histological tumor type on survival was significantly modulated by nodal status, meaning that patients with ILC tumors demonstrated better outcomes than patients with NST tumors when there was no or only limited nodal involvement (pN0/pN1), while outcomes were worse for patients with ILC compared to NST when many lymph nodes were affected (pN2/pN3). This effect was consistent over all four survival endpoints and independent of other prognostic factors. Importantly with regard to decision making in clinical practice, this was also true specifically for the group of patients with hormone receptor positive tumors and high nodal involvement (pN2/pN3): univariable and adjusted multivariable hazard ratios of ILC vs NST were 1.66 (95% CI 1.22–2.25, *p* = 0.001) and 1.55 (95% CI 1.10–2.19, *p* = 0.011) for DFS, 1.99 (95% CI 1.36–2.91, *p* < 0.001) and 1.75 (95% CI 1.14–2.70, *p* = 0.011) for OS, 1.98 (95% CI 1.30–3.02, *p* = 0.001) and 1.79 (95% CI 1.11–2.90, *p* = 0.018) for BCSS, and 1.44 (95% CI 1.00–2.08, *p* = 0.050) and 1.44 (95% CI 0.96–2.15, *p* = 0.079) for DDFS.

## Discussion

ILC is the second most common invasive breast cancer subtype at 10–15% of breast cancers and behaves clinically and biologically differently from NST. In our retrospective study, ILC was compared with NST based on pooled data from the three large prospective adjuvant breast cancer trials, SUCCESS A, B, and C, where intermediate-to-high-risk patients were treated with chemotherapy as well as endocrine and/or HER2-targeted therapy, depending on HR status and HER2neu status. The main focus of this study was on the comparison of clinicopathological characteristics as well as long-term outcome between the two histological tumor types, with special attention to a possible interaction effect between histological tumor type and nodal status as one of the main determinants of recurrence risk.

Several differences in demographic and tumor characteristics between ILC and NST were observed in our study, mostly consistent with the literature. On average, ILC patients were older than NST patients. They were also more likely to exhibit prognostically favorable tumor features, such as hormone receptor positive tumors with lower histological grading (predominantly luminal A-like tumors) than patients with NST [3, 6, 8, 36–39]. While our study confirms that ILC tends to be of the luminal type more often than NST, we nevertheless found higher rates of HER2 + ILC than other studies [40]. However, ILC tumors also had a higher tumor and nodal stage, both of which are unfavorable prognostic factors. This may be due at least partly to the poor imaging sensitivity of ILC, leading to delayed detection [2, 3, 7–10]. Accordingly, our study results show that ILC patients were more likely to receive mastectomy than NST patients, which is also in agreement with other published findings [3, 39].

There are conflicting comparative data on long-term survival of ILC and NST. Some studies have reported a poor prognosis for ILC compared to NST tumors and attributed this difference in outcome to larger tumor size, multifocality, and higher rates of lymph node metastasis in ILC tumors [3, 39, 41]. A few studies have found better survival for patients with ILC than for patients with NST [5, 22, 42], while other studies could not show a significant difference in survival between ILC and NST [37, 43]. These different results with regard to outcome comparisons between ILC and NST may be caused by the fact that ILC represents a very heterogeneous group of tumors with survival largely depending on the histological variant [40].

Our study revealed no significant survival differences (DFS, OS, DDFS, and BCSS) between ILC and NST carcinomas, independently of whether univariable or multivariable analyses adjusted for other prognostic factors were performed. All patients in the SUCCESS trials received adjuvant chemotherapy with FEC-Doc or cyclophosphamide, and patients with hormone receptor-positive tumors received antihormonal treatment with tamoxifen or an aromatase inhibitor. It is possible that, in the overall cohort, the prognostically favorable characteristics of ILC tumors (mostly HR-positive, low histological grade) and the protective effect of antihormonal treatment [11, 41] observed particularly in ILC tumors were offset by the prognostically unfavorable larger tumor size and higher lymph node involvement, resulting in similar outcomes to NST tumors.

However, while we found no difference in outcome between ILC and NST when all patients were analyzed together, our subgroup analyses according to nodal stage revealed an important interaction effect. Patients with ILC had better survival than patients with NST tumors when they had no or only limited nodal involvement (pN0/pN1), but had worse survival than patients with NST tumors when they had higher nodal involvement (pN2/pN3).

There are other large studies (Adachio, Chen, Yang, Zhao) that investigated potential differential survival between ILC and NST according to various risk factors. In a retrospective study of 1661 luminal NST and 104 luminal ILC patients, Adachi et al. showed that the prognosis of luminal ILC was significantly worse than that of luminal NST. The 5-year DFS was 91.9% (NST) vs. 88.4% (ILC) (*p* = 0.008) and the 5-year OS was 97.6% (NST) vs. 93.1% (ILC) (*p* = 0.030). However, while ILC was also associated with worse DFS in a multivariable analysis adjusted for other prognostic factors (*p* = 0.009), there was no significant effect of histological type on OS after adjustment for other prognostic factors (*p* = 0.262). Stratification by tumor size revealed a tendency for worse DFS in ILC compared to NST for larger (T3) tumors (26.7% ILC vs. 74.9% NST, *p* = 0.07). In node-positive patients with luminal disease, both 5-year DSF (77.4% vs. 85.5%, *p* = 0.02) and 5-year OS (83.3% vs. 94.4%, *p* = 0.017) were significantly worse in ILC compared to NST [8].

In a large SEER (Surveillance, Epidemiology, and End Results)-based retrospective study comprising 796,335 breast cancer patients (85,048 ILC and 711,287 NST) diagnosed between 1990 and 2013, Chen et al. demonstrated that the NST patients had better disease-specific survival (DSS) than ILC (HR 0.809; *p* < 0.0001). With regard to OS, they found a time-dependent effect (absent for DSS), with better survival for ILC patients before 5 years and better survival for NST after 5 years [3]. Furthermore, Chen et al. investigated whether the effect of histological type on survival differed by estrogen receptor (ER) and progesterone receptor (PR) status. Their results showed significantly better OS and DSS for NST in patients with both ER-positive/PR-positive and ER-positive/PR-negative tumors and no significant differences in OS and DSS between NST and ILC in patients with ER-negative/PR-positive tumors. However, one limitation of this study was the lack of information on adjuvant treatment modalities, which affects study interpretation.

In another large SEER-based retrospective study, Yang et al. identified 288,216 patients with NST and 30,190 patients with ILC diagnosed from 2006 to 2016. Using a propensity score matching method, they created two cohorts of patients with NST and ILC with 29,199 patients per cohort that were matched according to age, histological grade, tumor stage, nodal stage, ER status, PR status, surgery type, chemotherapy, and radiation therapy. Overall survival analyses were then conducted on these two matched cohorts [37]. The researchers found no difference in OS between NST and ILC tumors for the overall cohort. However, subgroup analyses revealed significantly worse OS for ILC compared to NST in high-risk patients with hormone receptor negative tumors (HR 1.26; 95% CI 1.01–1.58; *p* = 0.040) and with N2/N3 tumors (HR 1.15; 95% CI 1.04–1.27; *p* = 0.007), while no significant differences in OS were found for the corresponding low-risk groups of patients with hormone receptor positive tumors or N0/N1 tumors. Furthermore, hormone receptor-negative patients on CHT had worse OS in the ILC group than those in the NST group (HR 1.47; 95% CI 1.09–1.97; *p* = 0.010), whereas hormone receptor positive patients on CHT had similar OS in both groups (HR 0.99; 95% CI 0.91–1.09; *p* = 0.871). No significant differences in OS between the ILC and NST group were found in patients who did not require CHT irrespective of hormone receptor status.

In yet another retrospective SEER-based analysis including 171,881 patients, Zhao et al. examined the effect of molecular subtypes on prognosis of patients with ILC, NST, or mixed invasive ductal and lobular carcinoma. Multivariable analyses showed that, in the overall cohort, ILC was associated with better OS (HR 0.84; 95% CI 0.77–0.91; *p* < 0.001) but not with better breast cancer-specific survival (HR 0.92; 95% CI 0.82–1.02; *p* = 0.114) compared to NST. Subgroup analyses according to molecular subtypes adjusted for other factors showed significantly better OS in ILC patients compared to NST patients in the prognostically favorable HR(+)/HER2(−) subgroup, while there were no significant differences in OS between ILC and NST, in the other subgroups of patients with HR(+)/HER2(+), HR(−)/HER2(+) and HR(−)/HER2(−) tumors [22].

While the different results regarding the comparison of survival between ILC and NST in the overall cohorts of these studies add to the inconsistent data reported in the literature (see above), an interesting and clinically important pattern appears to emerge from these studies. There seems to be a tendency for better survival of ILC compared to NST for patients at low risk of recurrence (luminal breast cancer, smaller tumor size, no or limited nodal involvement), while patients with ILC tend to have worse survival than patients with NST when they are at intermediate-to-high risk of recurrence, as assessed by negative hormone receptor status, large tumor size or many involved lymph nodes. However, as no interaction effects are reported in any of these studies, there is a lack of statistically valid confirmation of a differential effect of the risk factors investigated on survival of patients with ILC or NST tumors.

To the best of our knowledge, our study is the first to report a consistent significant interaction effect between histological tumor type (ILC, NST) and nodal stage (pN0/pN1, pN2/pN3) on outcome of early breast cancer patients, thus confirming a differential effect of nodal stage on survival in patients with ILC or NST breast carcinomas. We have shown that patients with ILC tumors have a better prognosis than patients with NST tumors if they are at low risk of recurrence as determined by nodal stage pN0 or pN1, while the reverse is true for patients at high risk of recurrence based on their nodal stage (pN2/pN3). The 2-way interaction terms between histological type and nodal stage were significant in adjusted multivariable analyses for all four survival endpoints investigated (DFS, OS, BCSS, and DDFS).

A possible explanation for these risk-dependent differences in survival between ILC and NST could be that ILC seems to be less responsive to (neo-)adjuvant chemotherapy than NST [8, 12, 39, 44–47]. At the same time, there are data indicating that ILC is more responsive to adjuvant endocrine therapy with aromatase inhibitors than NST [16–18]. As patients with a high recurrence risk are more likely to receive (neo-)adjuvant chemotherapy and patients with a low risk of recurrence are more likely to receive only endocrine-based therapies, such a differential responsiveness of ILC and NST tumors to adjuvant risk-adapted therapies could at least partly explain our results.

One of our study’s strengths is the homogeneous patient sample from three large prospectively randomized adjuvant clinical breast cancer trials, which reduces the potentially confounding effects of heterogeneous patient samples and different treatment regimens. Furthermore, all data entries were checked for correctness and quality-controlled by the CRO during the SUCCESS trials, ensuring reliable and high-quality data. However, given the homogeneity of the patient population and the fact that only patients with intermediate-to-high risk of recurrence were included, our results may not be extrapolated to all early breast cancer patients, particularly when other adjuvant chemotherapy regimens are used. Another limitation of our study is the classification of biological tumor subtypes based on histopathological features only. Furthermore, we cannot exclude the possibility that patients may have been misclassified as having received no endocrine therapy, as some patients received their endocrine therapy at their general practitioners or gynecologists’ practice without always informing the study center. Finally, the possible impact of histological variants of ILC tumors on survival could not be investigated, as information on histological variants of ILC tumors was not available for the SUCCESS trials.

## Conclusion

In conclusion, our study showed that there was no significant difference in DFS, OS, BCSS, and DDFS between ILC and NST patients in both univariable and multivariable analyses adjusted for other prognostic factors and covariates. However, subgroup analyses demonstrate that, for patients with pN2/pN3 tumors, the prognosis of ILC was worse compared with NST, while for patients without or with less than four lymph node metastases (pN0/pN1) ILC was associated with better survival than NST. These results add to the growing evidence, suggesting that ILC should be considered a separate disease and confirm the need for both further research and clinical trials designed exclusively for breast cancer patients with ILC. More specifically, our results suggest that patients with ILC and pN2/pN3 disease should be closely watched for early signs of chemotherapy resistance and limited treatment response, as they might potentially benefit from a switch of chemotherapy and targeted treatment options. Upfront (prior to surgery) antihormonal treatment may also provide further information on chemosensitivity.

## Data Availability

Anonymized data will be made available to other researchers, subject to approval of a formal data access request that includes a detailed description of the purpose/scientific rationale of the proposed project. Requests are reviewed by the SUCCESS study group Steering Committee and will be approved if the proposed projects have a sound scientific or patient benefit rationale. Data recipients are required to sign a formal data sharing agreement which describes the conditions for release and requirements for data transfer, storage, archiving, publication, and intellectual property.

## Appendix

See Tables 3 and 4.

**Table 3:** Inclusion criteria of the SUCCESS studies

SUCCESS A	SUCCESS B	SUCCESS C
Primary epithelial invasive breast carcinoma pT1-4, pM0	Primary epithelial invasive breast carcinoma pT1-4, M0	Primary epithelial invasive breast carcinoma pT1-4, pN0-3, pM0
	HER2neu positive tumor (IHC+++ or FISH+)	HER2neu-negative tumor (IHC neg. or 1+ or FISH neg)
Histological evidence of axillary lymph node metastases pN1-3 or nodal negative high-risk patients N0/X, defined as pT > 2 or histopathological grading 3 or age ≤ 35 or negative hormone receptor status	Histological evidence of axillary lymph node metastases pN1-3 or nodal negative high-risk patients N0/X, defined as pT > 2 or histopathological grading 3 or age ≤ 35 or negative hormone receptor status	Histological evidence of axillary lymph node metastases pN1-3 or nodal negative high-risk patients N0/X, defined as pT > 2 or histopathological grading 3 or age ≤ 35 or negative hormone receptor status
R0 resection of the primary tumor (resection margins free of invasive carcinoma tissue), maximum 6 weeks ago	R0 resection of the primary tumor (resection margins free of invasive carcinoma tissue), maximum 6 weeks ago	R0 resection of the primary tumor (resection margins free of invasive carcinoma tissue), no more than 6 weeks ago
Women older than 18 years	Women older than 18 years	Women older than 18 years
General condition ≤ 2 on the ECOG scale	General condition ≤ 2 on the ECOG scale	General condition ≤ 2 on the ECOG scale
Adequate bone marrow reserve: leukocytes ≥ 3.0 × 109/l and platelets ≥ 100 × 109/l	Adequate bone marrow reserve: leukocytes ≥ 3.0 × 109/l and platelets ≥ 100 × 109/l	Adequate bone marrow reserve: leukocytes ≥ 3.0 × 109/l and platelets ≥ 100 × 109/l
Alanine aminotransferase, aspartate aminotransferase, and alkaline phosphatase within 1.5 times the normal value of the respective reference laboratory	Alanine aminotransferase, aspartate aminotransferase, bilirubin, and alkaline phosphatase within 1.5 times the normal value of the respective reference laboratory	Alanine aminotransferase, aspartate aminotransferase, and alkaline phosphatase within 1.5 times the normal value of the respective reference laboratory
Ensure regular follow-up during the study period	Ensure regular follow-up during the study period	Ensure regular follow-up during the study period
Understanding of the study concept and written informed consent	Understanding of the study concept and written informed consent	Understanding of the study concept and written informed consent
R0 resection of the primary tumor (resection margins free of invasive carcinoma tissue), maximum 6 weeks ago	Use of an effective contraceptive method (Pearl index < 1) in women of childbearing age during therapy and for at least 6 months afterward. Intrauterine pessaries (copper IUD) or surgical tubal ligation are considered safe methods of contraception	Patient is willing to participate in telephone-based lifestyle intervention

**Table 4:** Exclusion criteria of the SUCCESS studies

SUCCESS A	SUCCESS B	SUCCESS C
Inflammatory breast carcinoma	Inflammatory breast carcinoma	Inflammatory breast carcinoma
Prior or concurrent therapy with other cytotoxic or antineoplastic drugs that were not included within the study protocol	Prior or concurrent therapy with other cytotoxic or antineoplastic drugs that were not included within the study protocol	Prior or concurrent therapy with other cytotoxic or antineoplastic drugs that were not included within the study protocol
Secondary carcinoma (other than in situ carcinoma of the uterine cervix or adequately treated basal cell carcinoma)	Secondary carcinoma (other than in situ carcinoma of the uterine cervix or adequately treated basal cell carcinoma)	Secondary carcinoma (other than in situ carcinoma of the uterine cervix or adequately treated basal cell carcinoma)
Manifest cardiac impairment (cardiomyopathy with reduced ventricular function (NYHA > II), arrhythmias requiring therapy affecting LVEF, recent myocardial infarction or angina pectoris within the last 6 months, hypertension not controlled by medication)	Manifest cardiac impairment (cardiomyopathy with reduced ventricular function (NYHA > II), arrhythmias requiring therapy affecting LVEF, recent myocardial infarction or angina pectoris within the last 6 months, hypertension not controlled by medication)	Manifest cardiac impairment (cardiomyopathy with reduced ventricular function (NYHA > II), arrhythmias requiring therapy affecting LVEF, recent myocardial infarction or angina pectoris within the last 6 months, hypertension not controlled by medication)
Any known hypersensitivity to docetaxel, epirubicin, cyclophosphamide, fluorouracil, gemcitabine, or any other study drug	Any known hypersensitivity to docetaxel, epirubicin, cyclophosphamide, fluorouracil, gemcitabine, or other study drugs. The contraindications, warnings, or precautions listed in the product information of the approved preparations must be observed	Any known hypersensitivity to docetaxel, epirubicin, cyclophosphamide, or any other study drug
Use of any study drug within the last 3 weeks prior to study inclusion	Use of any study drug within the last 3 weeks prior to study inclusion	Use of any study drug within the last 3 weeks prior to study inclusion
Previous treatment with bisphosphonates within the last 6 months	Patients who are pregnant or breastfeeding (contraception must be ensured in premenopausal women): Intrauterine devices, surgical sterilization or, only in hormone receptor negative breast cancer patients, oral, subcutaneous, or transvaginal non-estrogen containing contraceptives)	Patients who are pregnant or breastfeeding (safe contraception must be ensured for premenopausal women: IUD or sterilization)
Patients who are pregnant or breastfeeding (contraception must be ensured in premenopausal women): Intrauterine devices, surgical sterilization or, in hormone receptor negative breast cancer patients only, oral, subcutaneous, or transvaginal non-estrogen containing contraceptives)	Poorly controlled, uncontrolled or unstable diabetes mellitus	Diabetes mellitus type 1 or insulin-dependent diabetes mellitus type 2
Impaired renal function demonstrated by calculated creatinine clearance of ≤ 30 ml/min, calculated according to the Cockcroft-Gault formula		Significant disorders of absorption or digestive capacity that prohibit the use of the study diet
Existing dental complaints, mandibular, and dental inflammation or acute or pre-existing necrosis of the jaws. Exposed bones in the oral cavity, or of slow-healing wounds after dental treatment		Self-reported inability to walk one kilometer at any speed
Recent (6 weeks) or planned dental or jaw surgery (extractions, implants)		Cardiovascular, respiratory, or musculoskeletal disease or joint problems that prevent moderate physical activity. This does not include moderate arthritis
		Psychiatric disorders that make participation in the intervention impossible
		Patients who do not have sufficient command of the German language to understand the nature of the study or the intervention measures included

## Abbreviations

AI: Aromatase inhibitors
BCSS: Breast cancer-specific survival
BfArM: Bundesinstitut für Arzneimittel und Medizinprodukte
BMI: Body mass index
CHT: Chemotherapy
CI: Confidence interval
CRO: Clinical research organization
DDFS: Distant disease-free survival
DFS: Disease-free survival
Doc: Docetaxel
DocG: Docetaxel gemcitabine
ER: Estrogen receptor
FEC: Fluorouracil, epirubicin, cyclophosphamide
FISH: Fluoreszenz in situ Hybridisierung
HER2: Human epidermal growth receptor 2
HR: Hormone receptor, Hormone receptor
IDC: Invasive ductal carcinoma
ILC: Invasive lobular carcinoma
IV: Intravenous
NST: Non-special type carcinoma
OS: Overall survival
p.o.: Perioral
pN: Pathological nodal status
PR: Progesterone receptor
pT: Primary tumor stage
s.c.: Subcutaneous
SD: Standard deviation
SEER: Surveillance, Epidemiology, and End Results
STEEP: Standardized Definitions of Efficacy Endpoints
SUCCESS: Simultaneous study of docetaxel-based anthracycline-free adjuvant treatment evaluation as well as
UICC: Union internationale contre le cancer
WHO: World Health Organization
